# Author Correction: Mechanism of human Lig1 regulation by PCNA in Okazaki fragment sealing

**DOI:** 10.1038/s41467-023-35873-x

**Published:** 2023-01-11

**Authors:** Kerry Blair, Muhammad Tehseen, Vlad-Stefan Raducanu, Taha Shahid, Claudia Lancey, Fahad Rashid, Ramon Crehuet, Samir M. Hamdan, Alfredo De Biasio

**Affiliations:** 1 Leicester Institute of Structural & Chemical Biology and Department of Molecular & Cell Biology, University of Leicester, Lancaster Rd, Leicester, LE1 7HB UK; 2 Bioscience Program, Division of Biological and Environmental Sciences and Engineering, King Abdullah University of Science and Technology, Thuwal, 23955 Saudi Arabia; 3 CSIC-Institute for Advanced Chemistry of Catalonia (IQAC) C/ Jordi Girona 18-26, 08034 Barcelona, Spain

**Keywords:** Cryoelectron microscopy, Enzyme mechanisms

Correction to: *Nature Communications* 10.1038/s41467-022-35475-z, published online 20 December 2022

In this article the author name Ramon Crehuet was incorrectly written as Ramon Creuhet. The original article has been corrected.

