# Supplementary material for: Mechanism of human Lig1 regulation by PCNA in Okazaki fragment sealing
Source: Nat Commun. 2022 Dec 20;13:7833. doi: 10.1038/s41467-022-35475-z (PMC9767926; doi:10.1038/s41467-022-35475-z)
Supplement: Supplementary file 1 — Supplementary Information [file 41467_2022_35475_MOESM1_ESM.pdf]

## Supplementary Information

# Mechanism of human Lig1 regulation by PCNA in Okazaki fragment sealing

Blair, et al.

Includes:

Supplementary Figures 1-15
Supplementary Tables 1-2
Description of Supplementary Movie 1

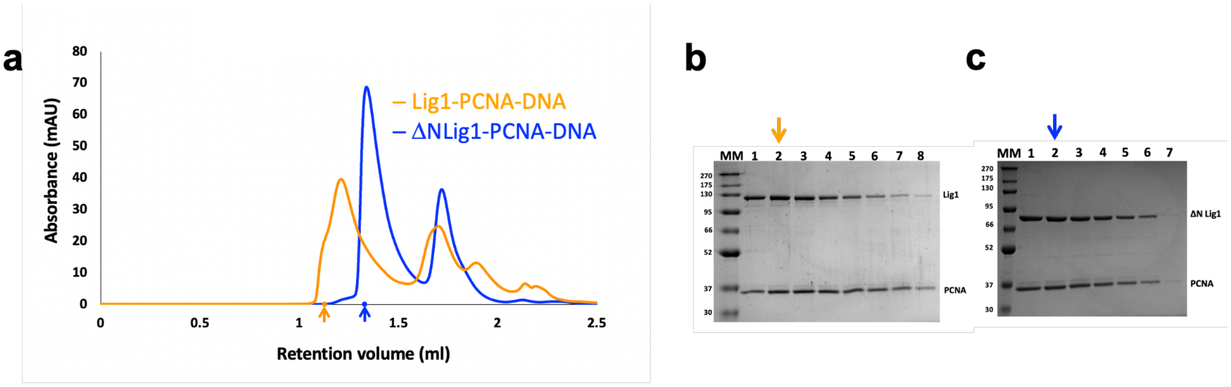


**Supplementary Figure 1**. **a)** Gel filtration chromatography of the Lig1–DNA–PCNA complex without ATP (orange trace) and the same complex with the N-terminal truncated Lig1 (ΔNLig1) (blue trace). **b)** Peak fractions from the orange trace in a) were analysed by SDS-PAGE. **c)** Peak fractions from the blue trace in a) were analysed by SDS-PAGE. Proteins corresponding to the bands are labelled on the right. Molecular weight standards are shown on the left. The fractions used for cryo-grid freezing are highlighted by orange and blue arrows.

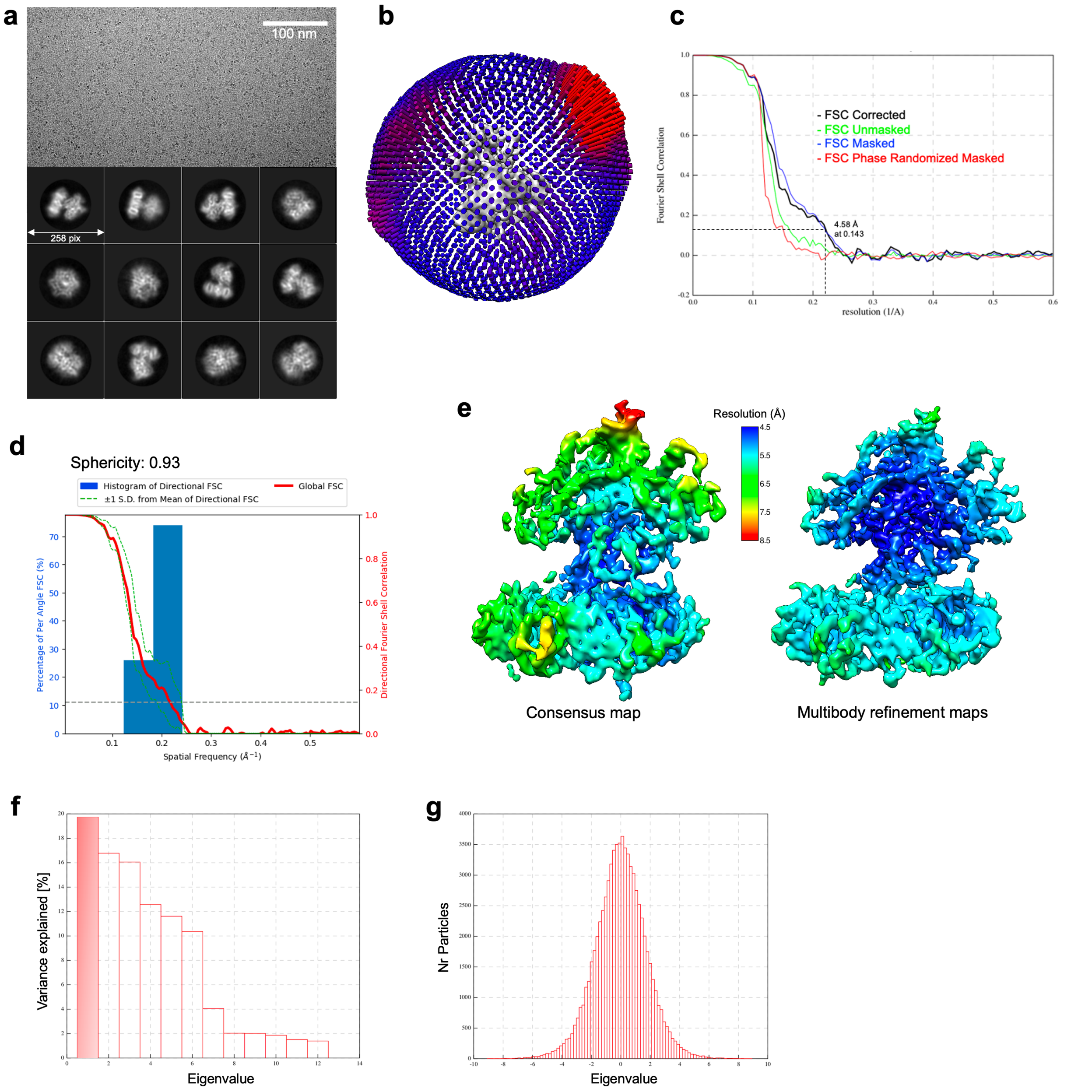


**Supplementary Figure 2.** *Cryo-EM of the Lig1–DNA–PCNA complex in the absence of ATP.* **a)** Electron micrograph (aligned sum) acquired using a Gatan K3 direct electron detector in super resolution mode, and representative 2D class averages. **b)** Angular distribution of projections. **c)** Gold-standard Fourier shell correlation, and resolution estimation using the 0.143 criterion. **d)** Map anisotropy analysis computed by 3DFSC[1]. **e)** Cryo-EM map colored by local resolution before and after multi-body refinement. **f)** Contribution of all eigenvectors to the variance in multi-body refinement, with the first eigenvector highlighted. **g)** Histogram of amplitudes along the first eigenvector in multi-body refinement. The histogram is unimodal, indicating continuous motion.

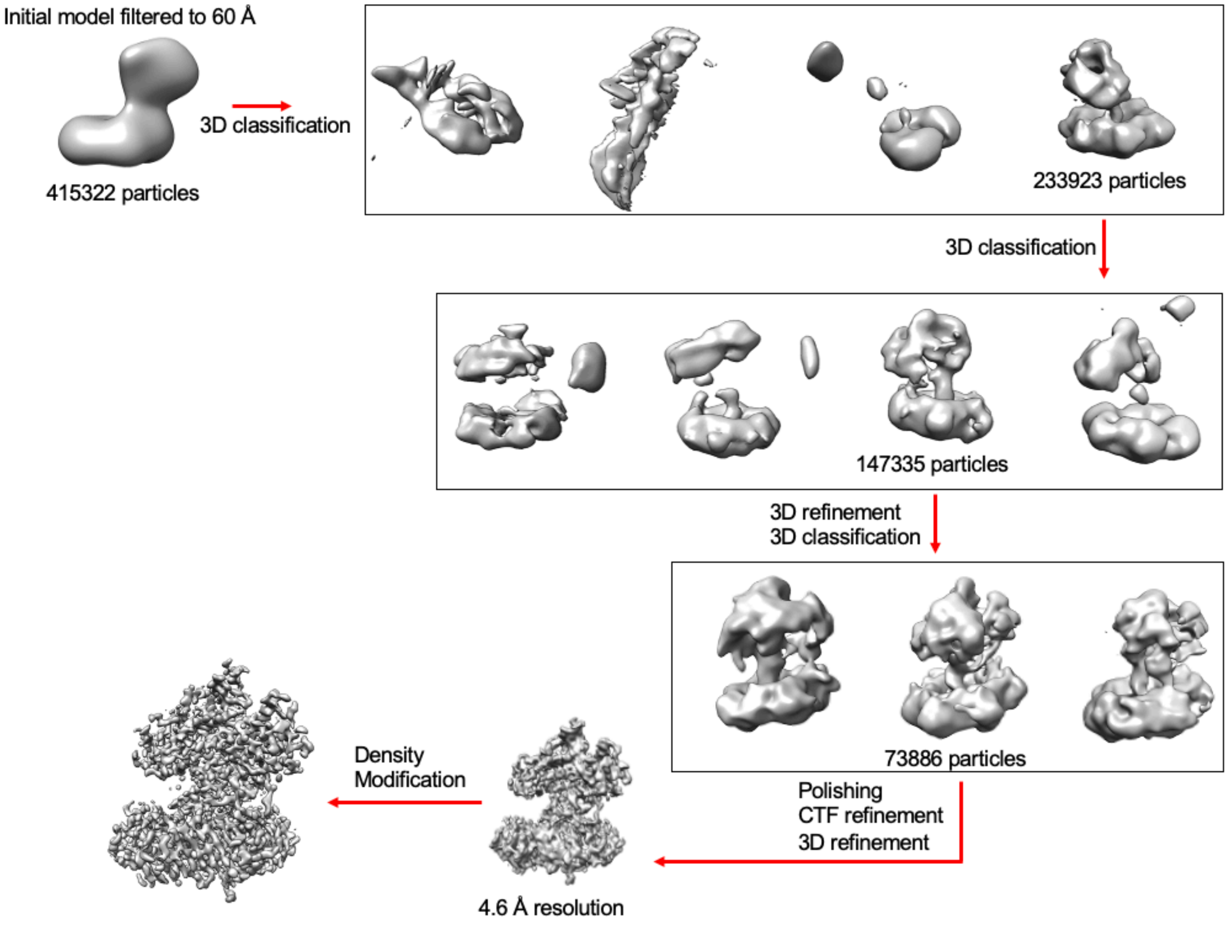


**Supplementary Figure 3.** Overview of image processing of the Lig1–DNA–PCNA complex in the absence of ATP.

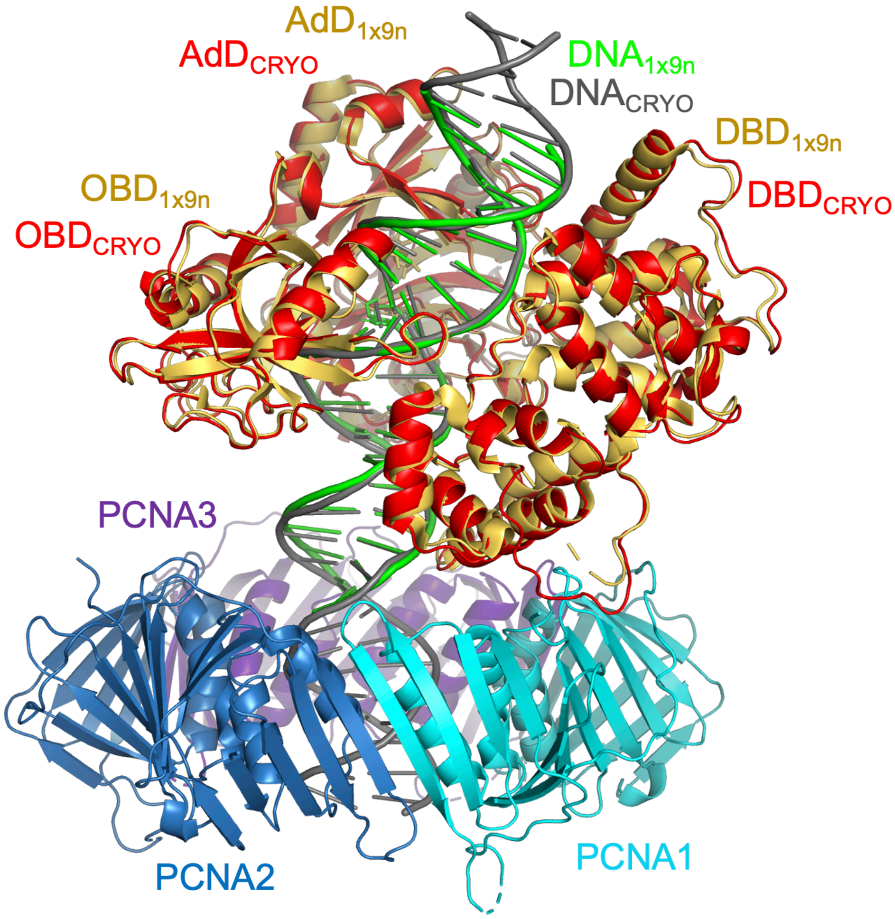


**Supplementary Figure 4.** *Cryo-EM structure of the Lig1–DNA–PCNA complex (CRYO) and X-ray structure of the Lig1–DNA complex (PDB: 1x9n), aligned on Lig1*. Structures are shown with ribbon representation, with domains labelled. This alignment shows the excellent correlation for both Lig1 and DNA components.

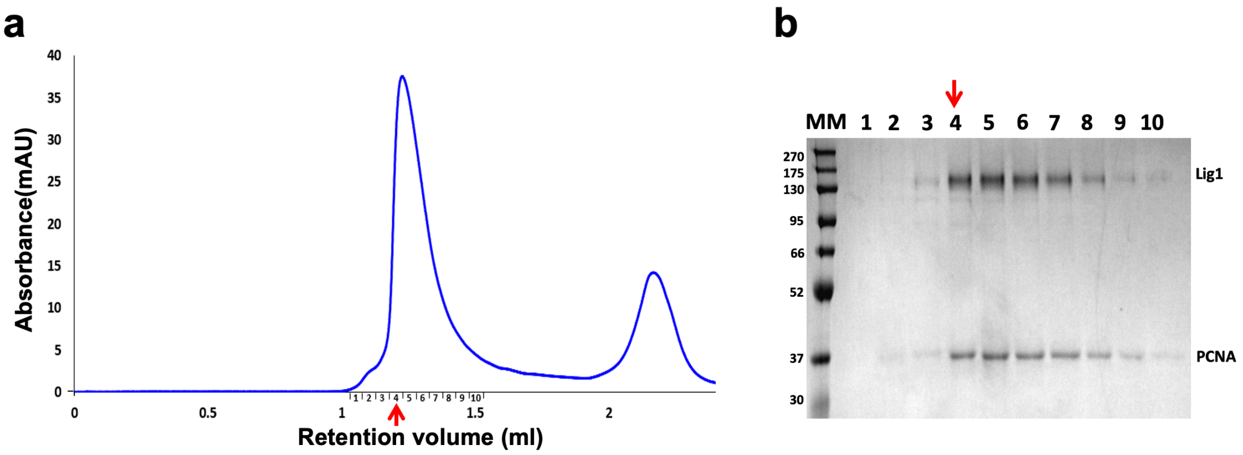


**Supplementary Figure 5**. *Sample separation of the Lig1–DNA–PCNA complex in the presence of ATP.* **a)** Gel filtration chromatography of the reconstituted complex. **b)** The numbered peak fractions in a) were analysed by SDS-PAGE (lanes 1-10). Proteins corresponding to the bands are labelled on the right. Molecular weight standards are shown on the left. The fraction used for cryo-grid freezing is highlighted by the red arrow.

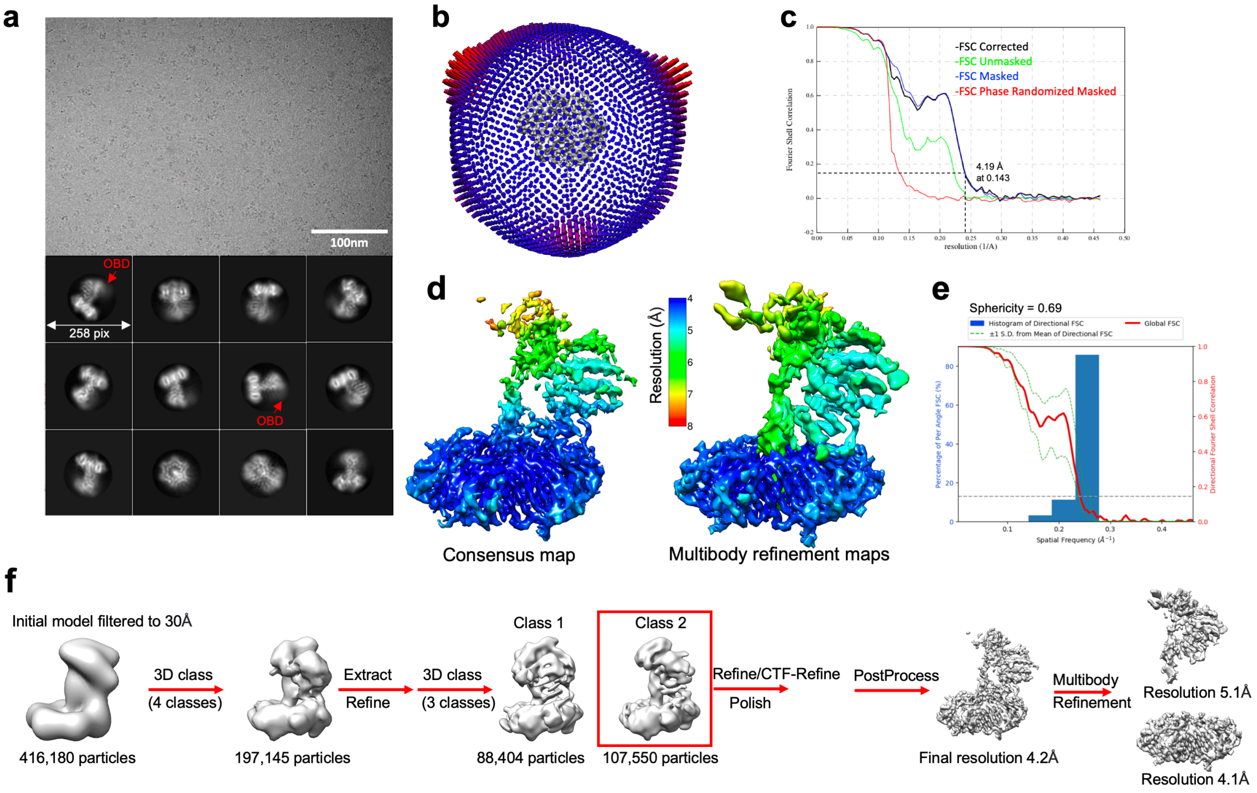


**Supplementary Figure 6.** *Cryo-EM of the Lig1–DNA–PCNA complex in open conformation*. **a)** Electron micrograph (aligned sum) acquired using a Gatan K3 direct electron detector in super resolution mode, and representative 2D class averages. The red arrow indicates the fuzzy density protruding from the Lig1 AdD assigned to the flexible OBD. **b)** Angular distribution of projections. **c)** Gold-standard Fourier shell correlation, and resolution estimation using the 0.143 criterion. **d)** Cryo-EM map colored by local resolution before and after multi-body refinement. **e)** Map anisotropy analysis for the consensus map computed by 3DFSC[1]. **f)** Overview of image processing.

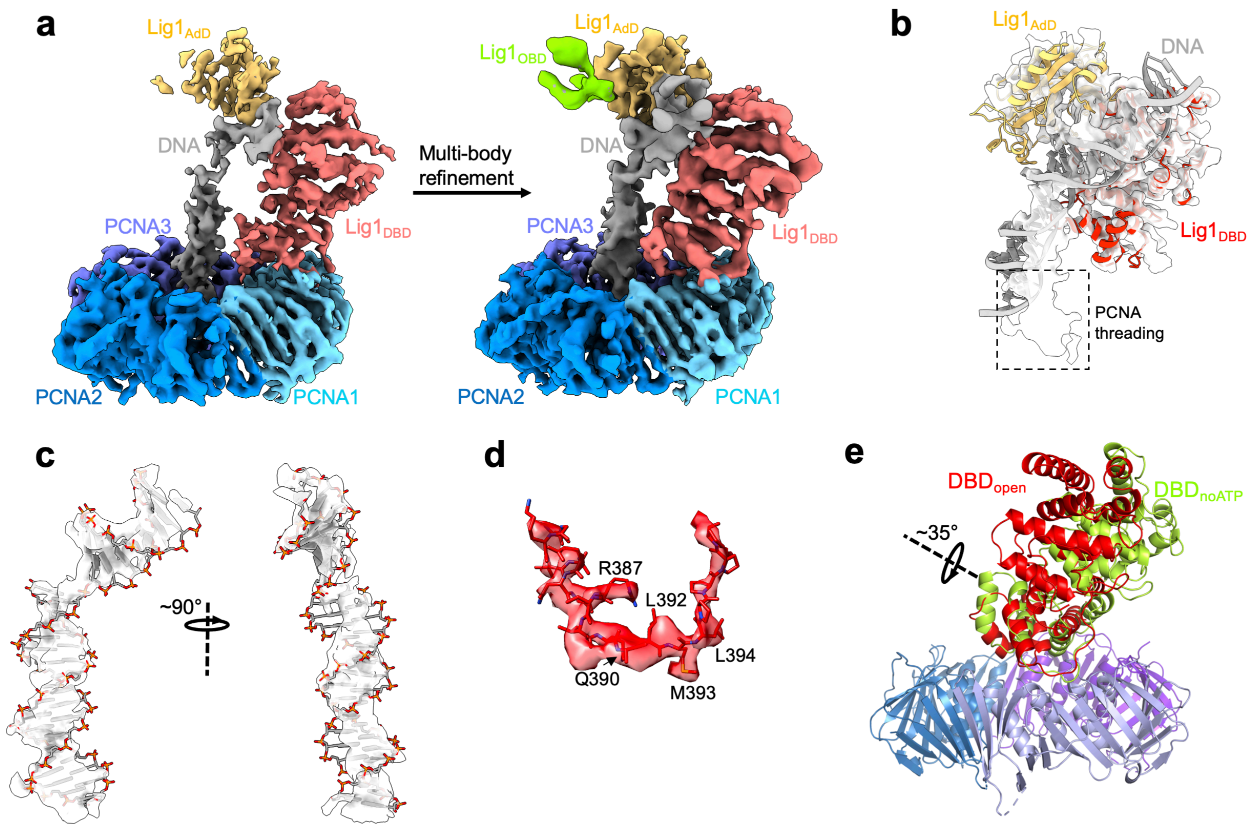


**Supplementary Figure 7.** *Cryo-EM structure of the open conformation of the Lig1–DNA–PCNA complex.* **a)** Cryo-EM map before and after multi-body refinement, colored by domains. **b)** Model of the Lig1-DNA complex reconstituted without ATP fitted into the Lig1-DNA body map of the open conformer from multi-body refinement. The OBD in the model was omitted for clarity. The dotted box lines the region of DNA threaded by PCNA in the open conformer, showing that the encircling of upstream DNA by PCNA forces the DNA to bend. **c)** Region of the multi-body refined map around the DNA, with model of bent DNA depicted in stick representation. **d)** Map region around the DBD PIP-box, with fitted models in stick representation. **e)** Structure of the open conformation and structure of the Lig1–DNA–PCNA complex obtained from reconstitution without ATP aligned on PCNA, showing that the positions of the ligase DBD domain in the two structures are related by a 35° rotation around the indicated axis. The DNA, AdD and OBD models are omitted for clarity.

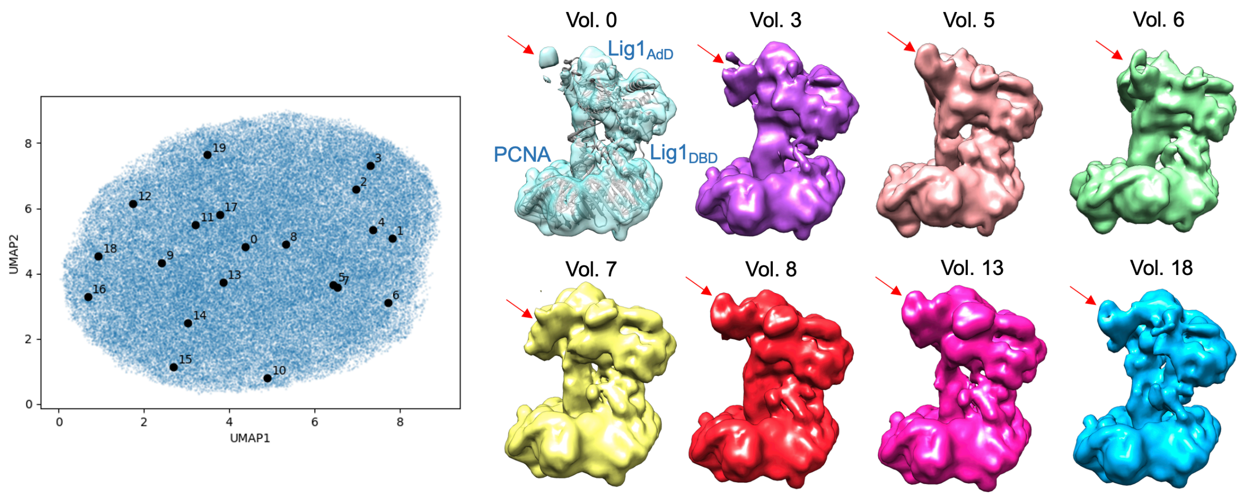


**Supplementary Figure 8.** *Particle heterogeneity analysis of the open conformation of the Lig1–DNA–PCNA complex*. In order to probe distinct conformational states of the ligase OBD, a particle heterogeneity analysis using CryoDRGN[2] was carried out. The figure shows the UMAP plot and exemplar volumes displaying density protruting from the AdD domain of Lig1 (highlighted with a red arrow), which is ascribed to the flexible OBD. Volumes were filtered by convoluting with a 3D Gaussian function of width equal to 2$\sigma$. A model of the Lig1–DNA–PCNA complex in open conformation was rigid-body fitted into Volume 0. In agreement with the 2D classification, multiple output reconstructions display globular density extending from the C-terminus of the AdD, pointing to different orientations of the OBD.

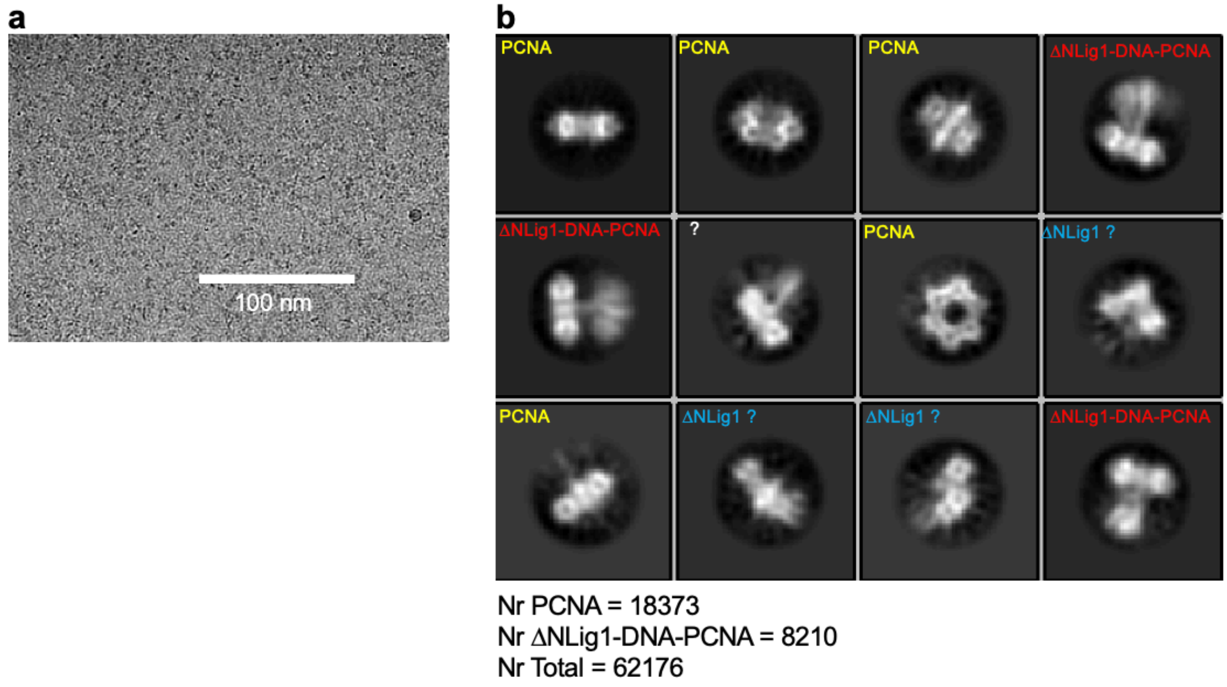


**Supplementary Figure 9.** *Cryo-EM analysis of formation of the ΔNLig1-DNA-PCNA complex* **a)** Electron micrograph (aligned sum) acquired using a Gatan K3 direct electron detector in super resolution mode. **b)** Representative 2D class averages and number of particles (Nr) ascribed to either free PCNA or ΔNLig1-DNA-PCNA complex, showing a low proportion of complex particles.

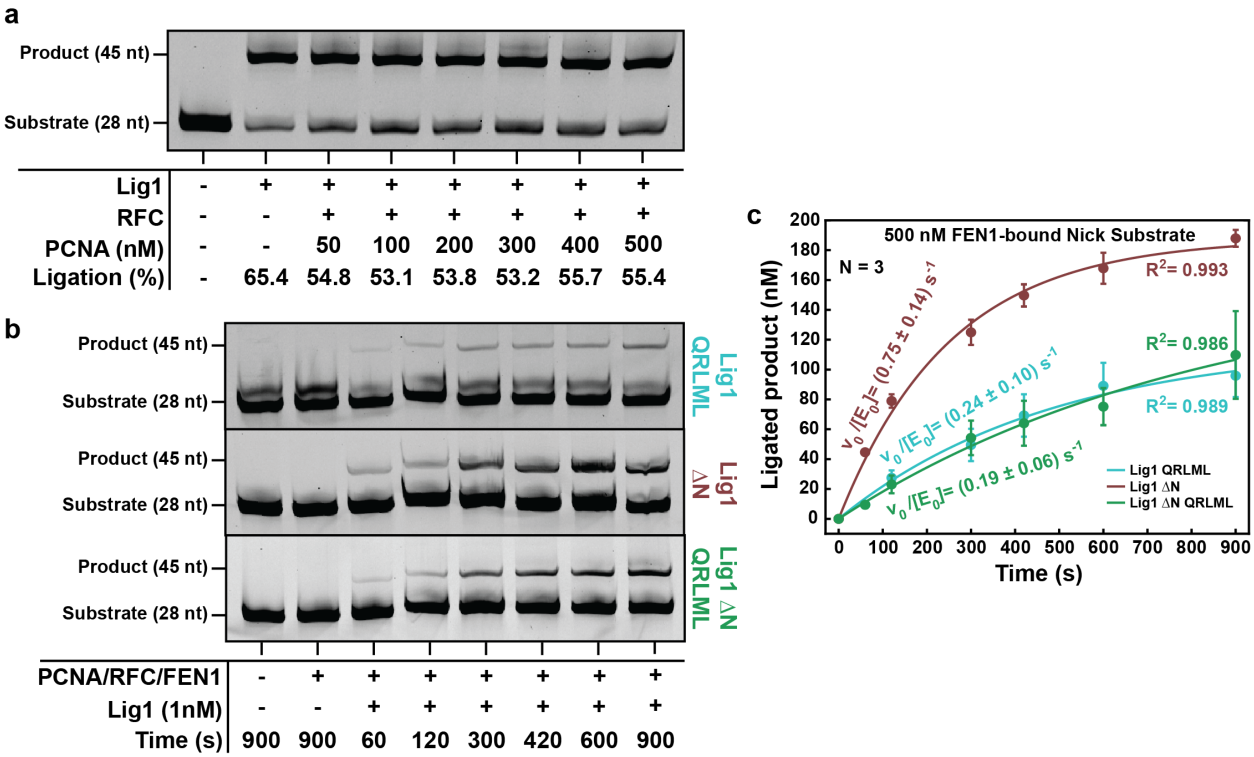


**Supplementary Figure 10.** *PCNA-dependent activity of Lig1 in absence and presence of FEN1*. **a)** A double-blocked (NeutrAvidin at both ends) DNA nick substrate (5 nM) was used for this experiment. PCNA (50-500 nM) was loaded on this substrate by the RFC clamp loader (40 nM). The reaction was initiated by the addition of Lig1 WT (1 nM). The reactions were incubated at 37°C for 15 minutes. The reactions were quenched by the addition of 40 mM EDTA. All reactions were incubated with Proteinase K at 50°C for 15 min and stopped by adding an equal volume of stop buffer (50 mM EDTA, 95% Formamide). DNA in the quenched reactions was denatured by heating at 95°C for 5 minutes and then immediately placed on ice. DNA reaction products were separated on 20 % denaturing Urea-PAGE gels and visualized using Typhoon Trio (GE Healthcare)**. b)** Multiple-turnover ligation by Lig1 variants in the presence of trapped PCNA and competitor FEN1. A double-blocked (NeutrAvidin bound to both DNA termini; 1 µM NeutrAvidin) DNA nick substrate (500 nM) was used for this experiment. PCNA (500 nM) was loaded on this substrate by RFC (500 nM). FEN1 D181A (500 nM) was added and incubated with the PCNA-loaded substrate. The reaction was initiated by adding each Lig1 variant (1 nM) and incubated for the indicated amount of time at 37°C. **c)** Quantification of the data from panel b) as described in the materials and methods section. For QRLML_Lig1 the burst parameters were A = (117.4 ± 27.2) nM and $\tau_{obs}$ = (485.7 ± 213.9) s. For ΔN_Lig1 the burst parameters were A = (187.9 ± 15.5) nM and $\tau_{obs}$ = (251.4 ± 57.0) s. For ΔN_QRLML_Lig1 the burst parameters were A = (161.9 ± 74.7) nM and $\tau_{obs}$ = (837.4 ± 577.0) s. The experimental data points represent the mean and one standard deviation of N=3 independent reactions. Source data are provided as a Source Data file.

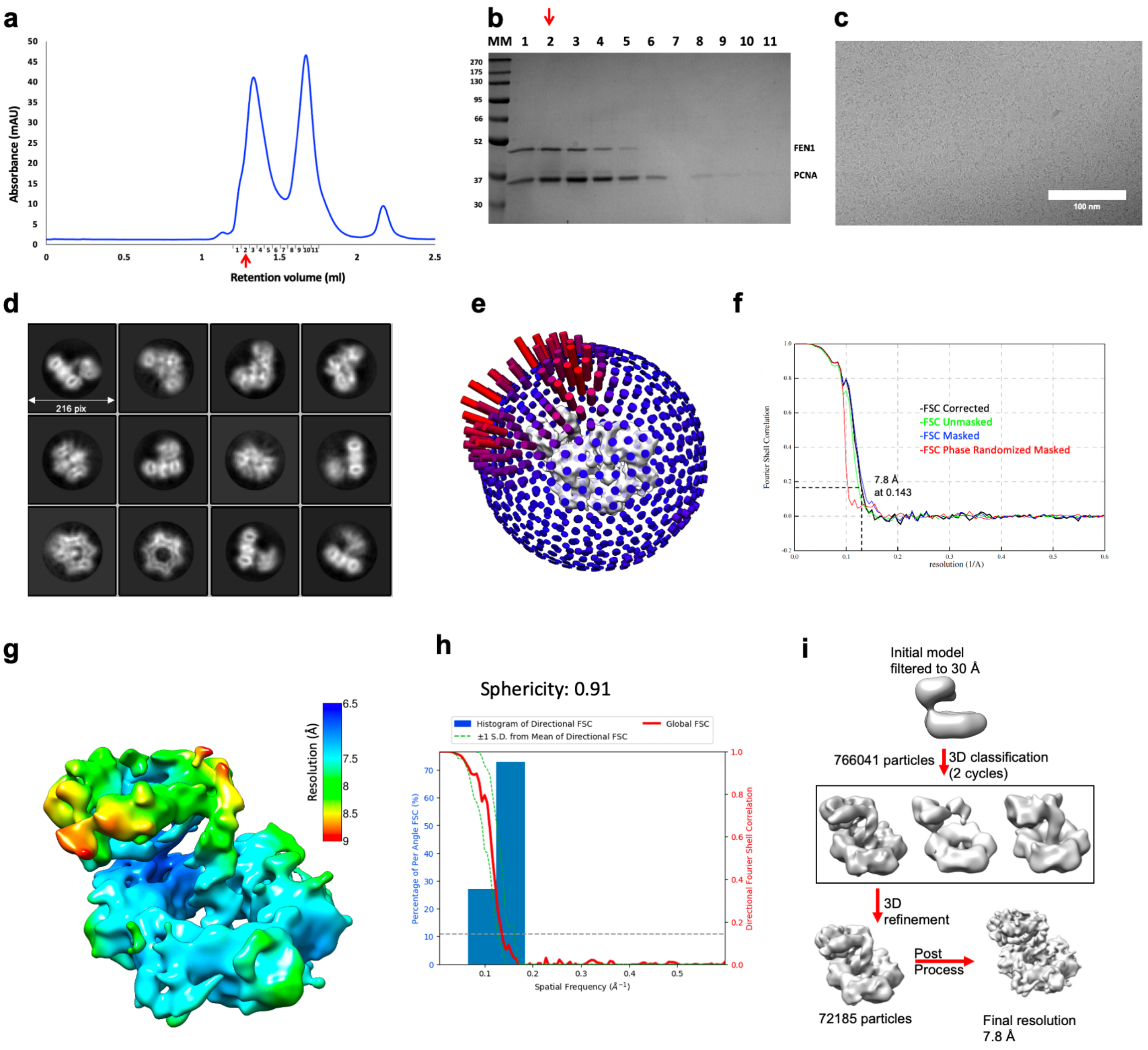


**Supplementary Figure 11.** *Cryo-EM of the FEN1–DNA–PCNA complex.* **a)** Gel filtration chromatography of the reconstituted complex. **b)** The numbered peak fractions in a) were analysed by SDS-PAGE (lanes 1–11). Proteins corresponding to the bands are labelled on the right. Molecular weight standards are shown on the left. The fraction used for cryo-grid freezing is highlighted by the red arrow. **c)** Electron micrograph (aligned sum) acquired using a Gatan K3 direct electron detector in super resolution mode. **d)** Representative 2D class averages. **e)** Angular distribution of projections. **f)** Gold-standard

Fourier shell correlation, and resolution estimation using the 0.143 criterion. **g)** Cryo-EM map colored by local resolution. **h)** Map anisotropy analysis computed by 3DFSC[1]. **i)** Overview of image processing.

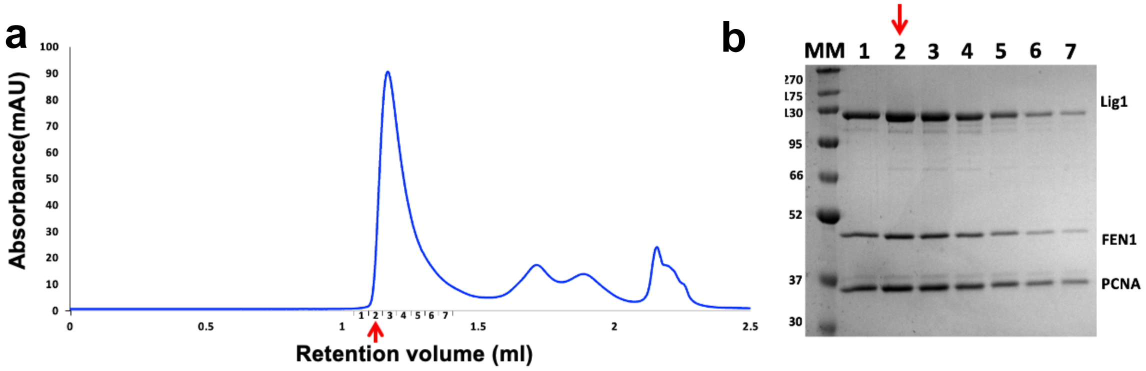


**Supplementary Figure 12**. *Sample separation of the Lig1–DNA–PCNA–*FEN1 *complex.* **a)** Gel filtration chromatography of the reconstituted complex. **b)** The numbered peak fractions in a) were analysed by SDS-PAGE (lanes 1-7). Proteins corresponding to the bands are labelled on the right. Molecular weight standards are shown on the left. The fraction used for cryo-grid freezing is highlighted by the red arrow.

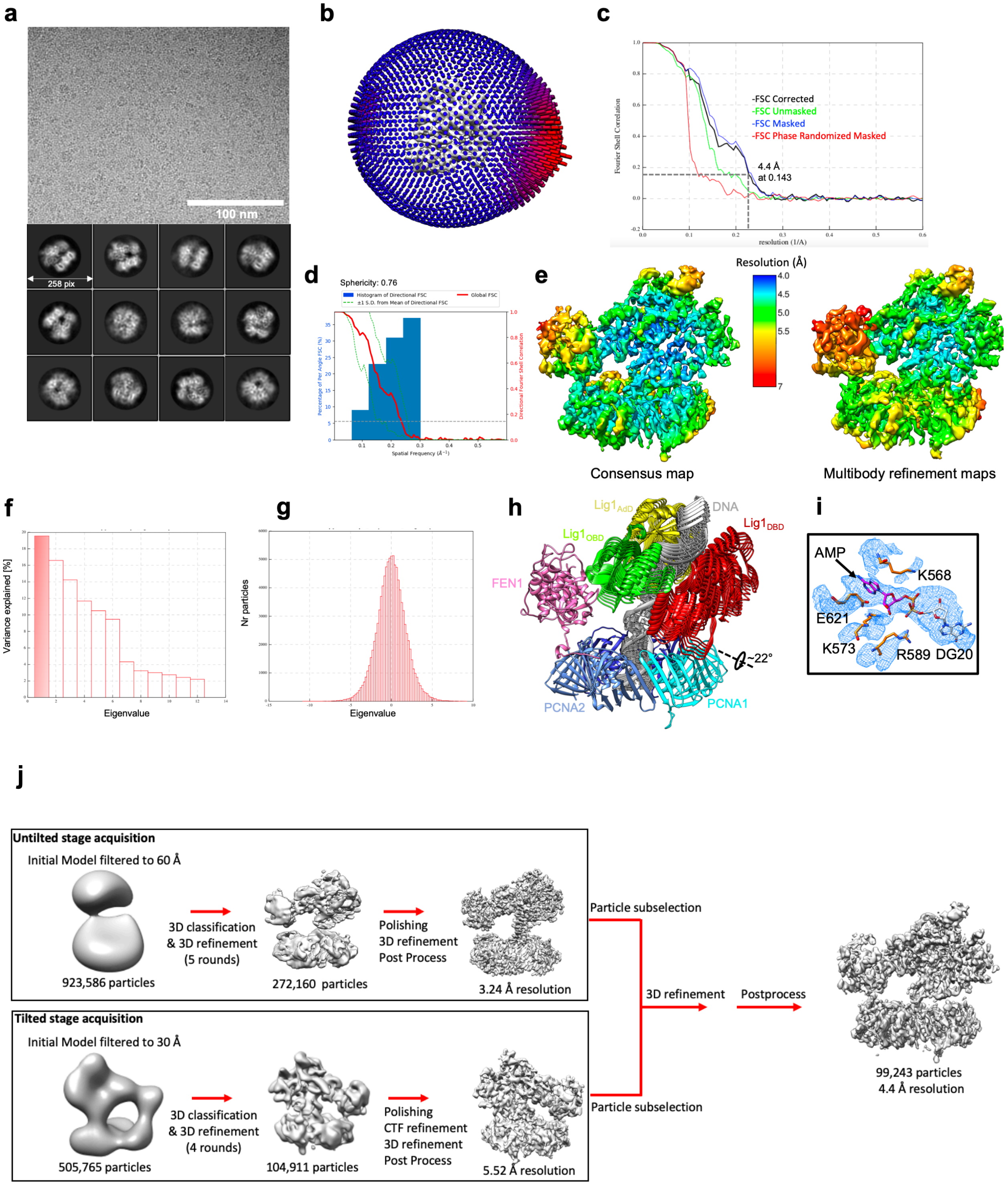


**Supplementary Figure 13.** *Cryo-EM of the Lig1–DNA–PCNA–*FEN1 *complex.* **a)** Electron micrograph (aligned sum) acquired using a Gatan K3 direct electron detector in super resolution mode, and representative 2D class averages. **b)** Angular distribution of projections. **c)** Gold-standard Fourier shell correlation, and resolution estimation using the 0.143 criterion. **d)** Map anisotropy analysis computed by 3DFSC[1]. **e)** Cryo-EM map

colored by local resolution before and after multi-body refinement. **f)** Contribution of all eigenvectors to the variance in multi-body refinement, with the first eigenvector highlighted. **g)** Histogram of amplitudes along the first eigenvector in multi-body refinement. The histogram is unimodal, indicating continuous motion. **h)** Motion represented by the first eigenvector from multi-body analysis. The first vector represents a motion involving a ~22° rotation of the Lig1-DNA body around the indicated axis. Five positions of the Lig1-DNA body spanning the full motion are shown. This mobility suggests that Lig1 and FEN1 do no stably interact in the toolbelt. **i)** Details of the cryo-EM map region at the Lig1 active site, with protein and DNA residues shown as sticks. **j)** Overview of image processing of the *Lig1–DNA–PCNA*–FEN1 complex.

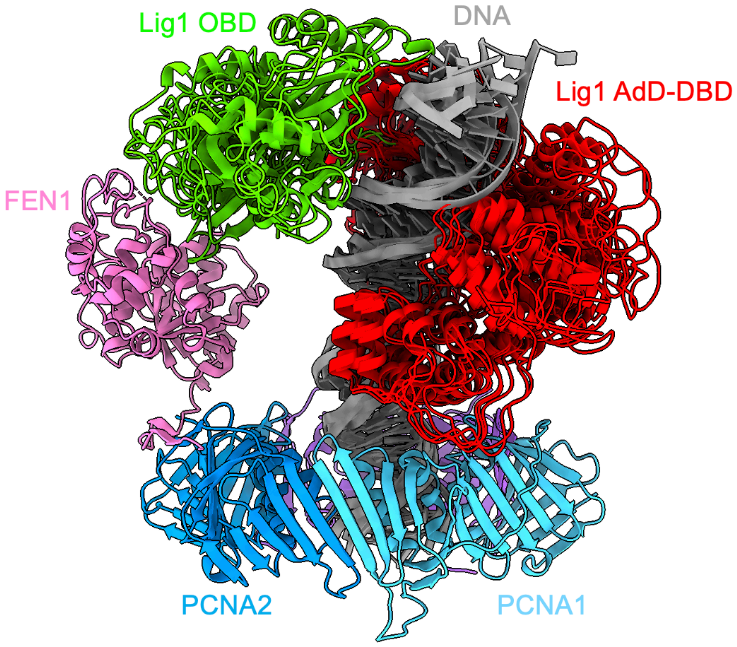


**Supplementary Figure 14.** *MD simulation to probe Lig1 OBD conformational space.* A MD simulation of the Lig1–DNA–PCNA complex was performed imposing a restrained distance between the OBD and DNA nick of 5.5 nm. The figure shows five extracted MD frames corresponding to the most divergent positions of the OBD superposed to Lig1 in the Lig1–DNA–PCNA–FEN1 structure. The PCNA component of the MD frames were deleted for clarity. The absence of clashes between OBD and FEN1 supports that FEN1 does not restrict the conformational space of the OBD.

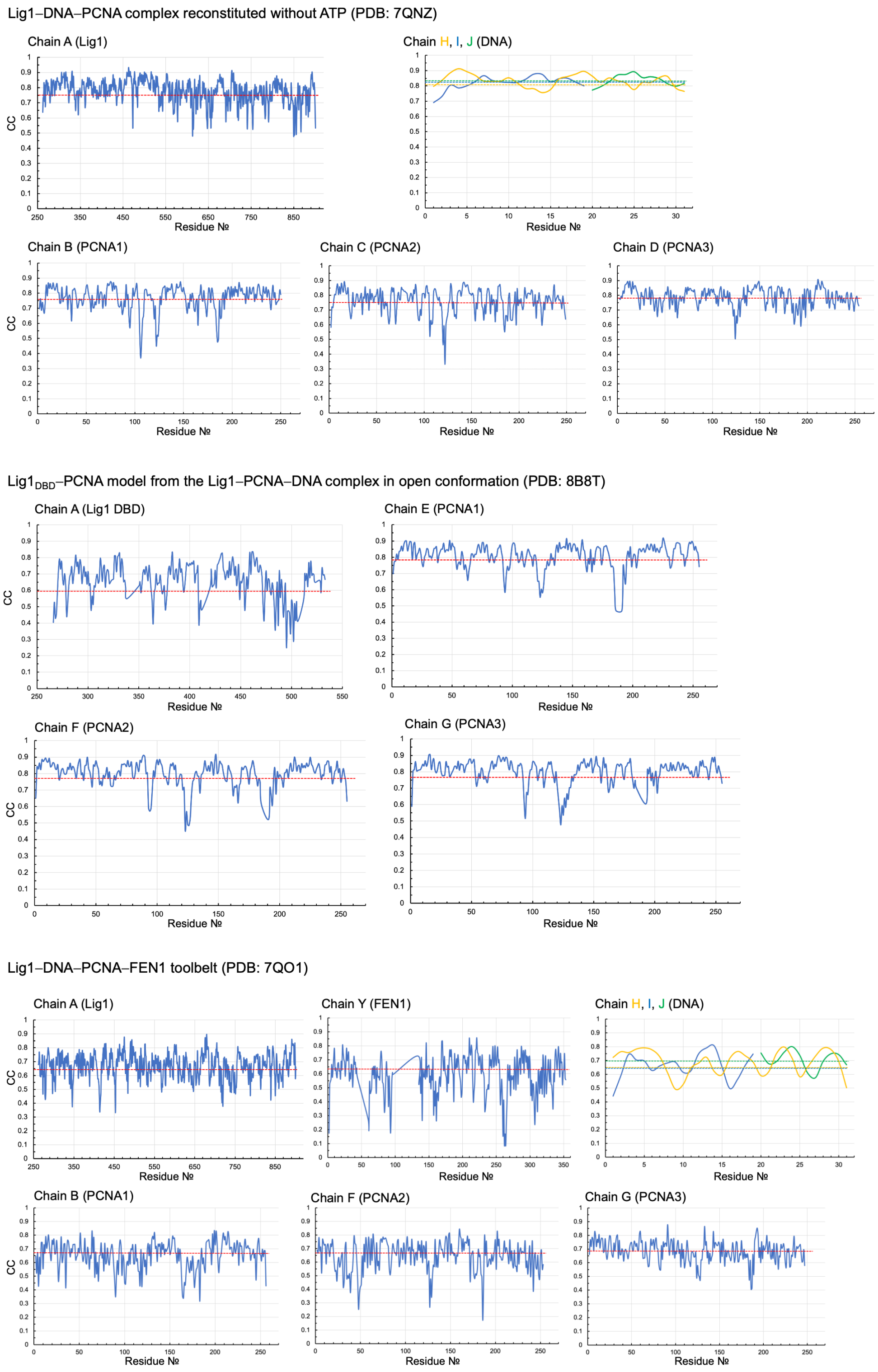


**Figure 15**. *Per-chain and per-residue map-to-model correlation coefficients (CC) versus residue number for the different structures included in the study*. The dotted lines represent the average chain correlation. The correlation coefficient for AMP in the Lig1-DNA-PCNA complex reconstituted without ATP and Lig1-DNA-PCNA-FEN1 toolbelt is 0.67 and 0.64, respectively.

**Supplementary Table 1. Cryo-EM data collection, refinement and validation statistics**

| | Lig1–DNA–PCNA without ATP (PDB:7QNZ) | Lig1–DNA–PCNA with ATP open conformer (PDB: 8B8T) | FEN1-DNA-PCNA | Lig1–DNA–PCNA–FEN1 toolbelt (PDB:7QO1) |
|---|---|---|---|---|
| **Data collection and processing** | | | | |
| Magnification | 105,000 | 81,000 | 105,000 | 105,000 |
| Voltage (kV) | 300 | 300 | 300 | 300 |
| Electron exposure (e–/Å$^2$) | 51.63 | 41.97 | 51.63 | 51.63 |
| Defocus range (μm) | -2.5 -to-1.0 | 2.0 to -0.8 | -2.5 to -1.0 | -2.5 to -1.0 |
| Pixel size (Å) | 0.835 | 1.086 | 0.835 | 0.835 |
| Symmetry imposed | C1 | C1 | C1 | C1 |
| Initial particle images (no.) | 415,322 | 2,988,615 | 766,041 | 1,904,204 |
| Final particle images (no.) | 73,886 | 107,550 | 72,185 | 99,243 |
| Map resolution (Å) | 4.58 | 4.19 | 7.8 | 4.40 |
| FSC threshold | 0.143 | 0.143 | 0.143 | 0.143 |
| Map resolution range (Å) | 4.39-7.16 | 4.02-18.8 | 6.74-8.74 | 4.13-7.36 |
| **Refinement** | | | | |
| Initial model used (PDB code) | 1AXC, 1X9N | 1AXC, 1X9N | 1UL1, 3Q8K | 1X9N, 1UL1 |
| Model resolution (Å) | N/A | N/A | N/A | N/A |
| FSC threshold | N/A | N/A | N/A | N/A |
| Model resolution range (Å) | N/A | N/A | N/A | N/A |
| Map sharpening B factor (Å$^2$) | Resolve cryo-EM | -136 | Anisotropic sharpening | -160 |
| Model composition | | | | |
| Non-hydrogen atoms | 11946 | 7181 | | 14179 |
| Protein residues | 1390 | 989 | | 1694 |
| Nucleotide residues | 62 | 0 | | 62 |
| Ligands | 1 | 0 | | 1 |
| B-factors (Å$^2$) | | | | |
| Protein | 136.56 | 69.30 | | 127.98 |
| Nucleotide | 168.44 | | | 150.17 |
| Ligand | 40.37 | | | 109.58 |
| R.m.s. deviations | | | | |
| Bond lengths (Å) | 0.012 | 0.011 | | 0.012 |
| Bond angles (°) | 1.874 | 1.872 | | 1.907 |
| Validation | | | | |
| MolProbity score | 1.53 | 1.15 | | 1.51 |
| Clashscore | 2.06 | 0.50 | | 2.24 |
| Poor rotamers (%) | 2.23 | 1.78 | | 1.51 |
| Ramachandran plot | | | | |
| Favored (%) | 95.77 | 95.85 | | 94.57 |
| Allowed (%) | 3.86 | 3.94 | | 4.25 |
| Disallowed (%) | 0.36 | 0.21 | | 0.18 |

## Supplementary Table 2. Oligonucleotides used in the biochemical assays

| | |
|---|---|
| Temp Lig1 Bio | 5’-BiotinTEG-TACCGAGCTCGAATTCGCCCGTTTCACGCCTGTTAGTTAATTCACTGGCCGTCGTTTTACAACGACGTGACTGGG-BiotinTEG-3’ |
| Lig1 5’ arm Cy5 | 5’-[Cy5] CCCAGTCACGTCGTTGTAAAACGACGGCCAGTGAATTA-3’ |
| Lig1 3’ arm Phos | 5’-[Phos] ACTAACAGGCGTGAAACGGGCGAATTCGAGCTCGGTA-3’ |
| Fluorescence Temp | 5’-TGACCGTTGTTTGACGGTCGTGAGGAGGAAAG/iAlex647N/TCCTCCTACGGCAG-3’ |
| Fluorescence 5’ arm | 5’-/5Phos/ ACGACCGTCAAACAACGGTCA/3BioTEG/-3’ |
| Fluorescence 3’ arm | 5’-CTGCCGTAGGAGGAACTTTCCTCCT/3ddC/-3’ |

## Supplementary Movie 1

MD trajectory of the Lig1–DNA–PCNA complex. Colour code: Lig1 DBD in red; Lig1 AdD in orange; Lig1 OBD in green; PCNA in blue.

## References


1. Zi Tan, Y. *et al.* Addressing preferred specimen orientation in single-particle cryo-EM through tilting. *Nature Methods* **14**, 793–796 (2017).
2. Zhong, E. D., Bepler, T., Berger, B. & Davis, J. H. CryoDRGN: reconstruction of heterogeneous cryo-EM structures using neural networks. *Nature Methods* **18**, 176–185 (2021).
