# Supplementary material for: Mechanism of human Lig1 regulation by PCNA in Okazaki fragment sealing
Source: Nat Commun. 2022 Dec 20;13:7833. doi: 10.1038/s41467-022-35475-z (PMC9767926; doi:10.1038/s41467-022-35475-z)
Supplement: Supplementary file 2 — Description of Additional Supplementary Files [file 41467_2022_35475_MOESM2_ESM.pdf]

**File Name:** Supplementary Movie 1

**Description:** MD trajectory of the Lig1-DNA-PCNA complex. Colour code: Lig1 DBD in red; Lig1 AdD in orange; Lig1 OBD in green; PCNA in blue
