# Supplementary material for: Mechanism of human Lig1 regulation by PCNA in Okazaki fragment sealing
Source: Nat Commun. 2022 Dec 20;13:7833. doi: 10.1038/s41467-022-35475-z (PMC9767926; doi:10.1038/s41467-022-35475-z)
Supplement: Supplementary file 4 — Reporting Summary [file 41467_2022_35475_MOESM4_ESM.pdf]

nature portfolio

Corresponding author(s): Alfredo De Biasio and Samir H. Hamdan

Last updated by author(s): December 1, 2022

Reporting Summary

Nature Portfolio wishes to improve the reproducibility of the work that we publish. This form provides structure for consistency and transparency in reporting. For further information on Nature Portfolio policies, see our Editorial Policies and the Editorial Policy Checklist.

Statistics

For all statistical analyses, confirm that the following items are present in the figure legend, table legend, main text, or Methods section.

n/a	Confirmed	
☐	☒	The exact sample size (*n*) for each experimental group/condition, given as a discrete number and unit of measurement
☐	☒	A statement on whether measurements were taken from distinct samples or whether the same sample was measured repeatedly
☒	☐	The statistical test(s) used AND whether they are one- or two-sided<br>*Only common tests should be described solely by name; describe more complex techniques in the Methods section.*
☒	☐	A description of all covariates tested
☒	☐	A description of any assumptions or corrections, such as tests of normality and adjustment for multiple comparisons
☐	☒	A full description of the statistical parameters including central tendency (e.g. means) or other basic estimates (e.g. regression coefficient) AND variation (e.g. standard deviation) or associated estimates of uncertainty (e.g. confidence intervals)
☒	☐	For null hypothesis testing, the test statistic (e.g. *F*, *t*, *r*) with confidence intervals, effect sizes, degrees of freedom and *P* value noted<br>*Give P values as exact values whenever suitable.*
☒	☐	For Bayesian analysis, information on the choice of priors and Markov chain Monte Carlo settings
☒	☐	For hierarchical and complex designs, identification of the appropriate level for tests and full reporting of outcomes
☒	☐	Estimates of effect sizes (e.g. Cohen's *d*, Pearson's *r*), indicating how they were calculated

Our web collection on statistics for biologists contains articles on many of the points above.

Software and code

Policy information about availability of computer code

Data collection	Cryo-EM data collection was performed using Thermofisher Scientific EPU 2.12 software
Data analysis	Cryo-EM data analysis was performed using Relion 3.1 software; Gel data were analysed with GelAnalyser v19.1; Structural models were analysed using Pymol v2.x, Coot v0.9.6, USFC Chimera v1.16 and Phenix v1.19.2; the AlphaFold model was built with ColabFold 1.3.0; MD models were analysed using MDTraj64 and scikit-learn. MD simulations were run in Gromacs 2020.6.

For manuscripts utilizing custom algorithms or software that are central to the research but not yet described in published literature, software must be made available to editors and reviewers. We strongly encourage code deposition in a community repository (e.g. GitHub). See the Nature Portfolio guidelines for submitting code & software for further information.

Data

Policy information about availability of data

All manuscripts must include a data availability statement. This statement should provide the following information, where applicable:

- Accession codes, unique identifiers, or web links for publicly available datasets
- A description of any restrictions on data availability
- For clinical datasets or third party data, please ensure that the statement adheres to our policy

The map of the Lig1-DNA-PCNA complex reconstituted without ATP has been deposited in the EMBD with accession code EMD-14078, and the atomic model in the Protein Data Bank under accession code PDB 7QNZ. The consensus and multi-body refined maps of the open conformation of the Lig1-DNA-PCNA complex reconstituted with ATP have been deposited in the EMBD with accession code EMD-15921, and the atomic model in the Protein Data Bank under accession code PDB 8B8T. The map of the FEN1-DNA-PCNA complex has been deposited with accession code EMD-15385. The map of the Lig1-DNA-PCNA-FEN1 complex has been deposited in the EMBD with accession code EMD-14080, and the atomic model in the Protein Data Bank under accession code PDB 7QO1.Correspondence and

requests for materials should be addressed to S.M.H. (samir.hamdan@kaust.edu.sa) or A.D.B. (alfredo.debiasio@kaust.edu.sa). Models included in this study were built using the following available structures: structure of the human Lig1-DNA-AMP complex (PDB ID 1X9N), structure of the PCNA homotrimer (PDB ID 1AXC), structure of PCNA and FEN1 (PDB ID 1UL1), structure of FEN1 bound to product nicked DNA (PDB ID 3Q8K).

Field-specific reporting

Please select the one below that is the best fit for your research. If you are not sure, read the appropriate sections before making your selection.

☒ Life sciences ☐ Behavioural & social sciences ☐ Ecological, evolutionary & environmental sciences

For a reference copy of the document with all sections, see nature.com/documents/nr-reporting-summary-flat.pdf

Life sciences study design

All studies must disclose on these points even when the disclosure is negative.

Sample size	For the Lig1-DNA-PCNA complex reconstituted without ATP, the Lig1-DNA-PCNA complex in open conformation, the Lig1-PCNA-DNA-FEN1 complex and for the FEN1-DNA-PCNA complex we collected 2941, 2540, 7647 and 3552 raw movie micrographs, respectively. The final map resolutions after processing of these micrographs were 4.6, 4.2, 4.4 and 7.8 angstroms. We deemed the sample size sufficient because we believe that the limitation in final resolution is due to complex partial flexibility, and that acquiring additional data could hardly improve resolution.
Data exclusions	No data was excluded from the bulk DNA ligation assays. Micrographs containing limited resolution information were discarded. Particles contributing to low 2D and 3D averages were also discarded.
Replication	FRET binding assays and Ligation assays were repeated 3 times, and repetitions were successful. Cryo-EM experiments rely on averaging of tens of thousands of particles and were highly reproducible.
Randomization	Randomization does not apply to the current study, which only includes Cryo-EM data, bulk DNA ligation assay data, binding and FRET data.
Blinding	Blinding does not apply to the current study, which only includes Cryo-EM data, bulk DNA ligation assay data, binding and FRET data.

Reporting for specific materials, systems and methods

We require information from authors about some types of materials, experimental systems and methods used in many studies. Here, indicate whether each material, system or method listed is relevant to your study. If you are not sure if a list item applies to your research, read the appropriate section before selecting a response.

Materials & experimental systems

n/a	Involved in the study	
☒	☐	Antibodies
☒	☐	Eukaryotic cell lines
☒	☐	Palaeontology and archaeology
☒	☐	Animals and other organisms
☒	☐	Human research participants
☒	☐	Clinical data
☒	☐	Dual use research of concern

Methods

n/a	Involved in the study	
☒	☐	ChIP-seq
☒	☐	Flow cytometry
☒	☐	MRI-based neuroimaging
